# Single-Source Thermal Evaporation Growth and the Tuning Surface Passivation Layer Thickness Effect in Enhanced Amplified Spontaneous Emission Properties of CsPb(Br_0.5_Cl_0.5_)_3_ Perovskite Films

**DOI:** 10.3390/polym12122953

**Published:** 2020-12-10

**Authors:** Saif M. H. Qaid, Hamid M. Ghaithan, Bandar Ali Al-Asbahi, Abdullah S. Aldwayyan

**Affiliations:** 1 Physics and Astronomy Department, College of Science, King Saud University, Riyadh 11451, Saudi Arabia; balasbahi@ksu.edu.sa (B.A.A.-A.); dwayyan@ksu.edu.sa (A.S.A.); 2 Department of Physics, Faculty of Science, Ibb University, Ibb 70270, Yemen; 3 Department of Physics, Faculty of Science, Sana’a University, Sana’a 12544, Yemen; 4 King Abdullah Institute for Nanotechnology, King Saud University, Riyadh 11451, Saudi Arabia; 5 K.A. CARE Energy Research and Innovation Center at Riyadh, Riyadh 11451, Saudi Arabia

**Keywords:** perovskite, surface passivation, PMMA, blue emission, amplified spontaneous emission, single-source thermal evaporation

## Abstract

High-quality inorganic cesium lead halide perovskite CsPb(Br_0.5_Cl_0.5_)_3_ thin films were successfully achieved through evaporation of the precursors and deposition sequentially by a single-source thermal evaporation system. The different melting points of the precursors were enabled us to evaporate precursors one by one in one trip. The resulting films through its fabrication were smooth and pinhole-free. Furthermore, this technique enabled complete surface coverage by high-quality perovskite crystallization and more moisture stability oppositely of that produce by solution-processed. Then the perovskite films were encapsulated by evaporated a polymethyl methacrylate (PMMA) polymer as a specialized surface passivation approach with various thicknesses. The blue emission, high photoluminescence quantum yield (PLQY), stable, and low threshold of amplified spontaneous emission (ASE) properties of CsPb(Br_0.5_Cl_0.5_)_3_ films in the bulk structure at room temperature were achieved. The effects of the surface-passivation layer and its thickness on the optical response were examined. Detailed analysis of the dependence of ASE properties on the surface passivation layer thickness was performed, and it was determined this achieves performance optimization. The ASE characteristics of bare perovskite thin film were influenced by the incorporation of the PMMA with various thicknesses. The improvement to the surface layer of perovskite thin films compared to that of the bare perovskite thin film was attributed to the combination of thermal evaporation deposition and surface encapsulation. The best results were achieved when using a low PMMA thickness up to 100 nm and reducing the ASE threshold by ~11 μJ/cm^2^ when compared with free-encapsulation and by ~13 μJ/cm^2^ when encapsulation occurs at 200 nm or thicker. Compared to the bare CsPb(Br_0.5_Cl_0.5_)_3_, ASE reduced 1.1 times when the PMMA thickness was 100 nm.

## 1. Introduction

Organic-inorganic (hybrid) and inorganic lead halide perovskite structures with monovalent A cations and X anions (or single and mixed versions) have prominent APbX_3_ (e.g., A = Cs, MA, and FA; X = Cl, Br, and I, respectively). Perovskite materials have significant potential for use in optoelectronic applications, including solar cells, photodetectors, and light-emitting devices (LEDs), although the potential applications for perovskite inorganic cations CsPbX_3_ perovskite quantum dots (PQDs) are oriented mainly toward LEDs, with few reports on applications in photodetectors and solar cells. CsPbX_3_ PQDs have a short exciton radiative lifetime, long carrier diffusion length, excellent charge transport properties, narrow emission linewidth, and high photoluminescence quantum yields (PLQYs) in addition to bandgap tunability and broad chemical compositions [1,2,3,4,5,6]. Compositionally tunable bandgap energies can be obtained by using a mixture of halide ion sources (i.e., Cl/Br and Br/I). Additionally, variations in the halide ions of the perovskite matrix strongly affect the structural and optical properties. However, due to the formation energies of various cesium lead halide alloys, theoretical calculations predict that the formation of the (Cl/I) mixed alloy is difficult compared with (Br/Cl) (or) (Br/I) mixtures. CsPbX_3_ exhibit relatively improved stability compared with those of their organic–inorganic hybrid equivalents (e.g., MAPbX_3_ and FAPbX_3_). However, the low formation energy of crystal lattices coupled with the high delocalization activity of surface ions makes CsPbX_3_ highly susceptible to polar solvents and sensitive to moisture and air, anion-exchange reactions, and thermal heating in practical operation [7,8,9,10]. Such inherent instability significantly impedes the further development and future application of CsPbX_3_ in optoelectronics fields. Therefore, it is essential to identify an effective method to improve stability. For this reason, several protective strategies have been proposed, including surface passivation. Surface passivation states and surface modifications have become an important research focus for practical applications due to having a significant impact on the stability and other physical properties of perovskite materials, including improved optical responses [11,12]. Additionally, surface passivation can involve encapsulation by organic polymer matrices (e.g., polymethyl methacrylate (PMMA) [13,14], polyvinylidene fluoride, ethylene vinyl acetate, or anthracene [15,16,17]). Furthermore, the surface passivation process can involve encapsulation by some mesoporous inorganic dielectric materials by embedding PQDs into mesoporous particles via a facile mixing method (e.g., SiO_2_ [17,18,19,20,21,22,23,24], TiO_2_ [25,26,27,28], or glass sheets [29]). The polymer films should be compatible with perovskite films under appropriate processing conditions, and encapsulation materials should be transparent to ensure that the optical properties are preserved [30,31]. Based on these requirements, PMMA polymers have been widely used as the material of choice in plastic optical fiber fabrication [30,32] and optoelectronic applications [33].

Previous studies have examined physical approaches to improving the light amplification efficiency of the active layer. For example, the approach by Li et al. [34] realized perovskite-PMMA bilayers with MAPbBr_3_ active films through a two-step sequential deposition process that was then coated with a PMMA top layer that was deposited by spin coating. The PMMA-coated film showed higher photoluminescence (PL) intensity, slower PL relaxation dynamics, and 2.2 times lower amplified spontaneous emission (ASE) threshold. A similar approach has also been reported for MAPbI3 films, which had a threshold reduction (approximately 1.5 times) due to the deposition of the PMMA top layer and a strong increase in the ASE intensity (up to 13.9 times) due to the further addition of a PMMA-gold nanorod layer, which was attributed to plasmonic effects [35]. Another group demonstrated thin films of cesium lead bromide, which showed a high photoluminescence quantum yield of 68% and ASE with a low threshold at room temperature [36]. 

There are additional advantages of using perovskites as a bulk structure when used at the nanostructure scale, such as in nanowires, nanotube, quantum dots. Moreover, in low-dimensional materials, the crystallinity is better than that of the materials that have a bulk structure due to the reduced trap density and enlarged mechanical stability of QDs. These properties may lead to greatly extended optoelectronic device longevity and are beneficial for flexible electronics. To date, the large dimensions of these crystals have made them ill-suited to form optoelectronic devices based on rectifying junctions. However, their potential applications in bulk semiconductor optical amplifiers and down-converters remain promising, and the prospect of a substrate-controlled growth of patterned perovskite single crystals is a topic of intense discussion. Therefore, many researchers have tried to render inorganic perovskite materials as a source for obtaining a medium for the perovskite laser and to disprove the current belief that efficient light emission from CsPbX_3_ perovskites can only be achieved with nanoparticles. Historically, producing perovskites with color-stable and efficient blue (below 480 nm) at room temperature remains challenging due to the high trap density as reported with this material, especially in limited light stability [37,38]. Therefore, halide exchange of Br/Cl is one widely employed strategy to achieve blue emission wavelength between 450 and 490 nm. We also foresee that our results will have an important impact on achieving blue light emissions from perovskite as a bulk structure.

In this study, the fabrication process involves the deposition of consecutive perovskite and PMMA films by thermal evaporation. Here, we highlighted the film preparation method through the fabrication technique vapor deposition, which produces smooth and pinhole-free thin films. In contrast, in solution-processed films, the precise influence of moisture could not be deconvoluted. To evaporate the precursor materials CsX and PbX_2_ (X = Br, Cl) by thermal vapor deposition, there are two main methods to create CsPbX_3_ perovskite thin film, namely, co-evaporation (precursor are evaporated simultaneously) and sequential method. In the co-evaporation method, it requires very careful control of deposition rates to ensure that the final thin film consists of the correct stoichiometry. In contrast, the sequential method—where precursors are deposited sequentially—is easier due to the step by step process. This study focuses on films produced by the single-source sequential thermal evaporation method. The thickness of each layer could be reliably controlled by injecting the weight of the polymer powder into the boat. Mainly, the focus of this study is to conduct an in-depth investigation and analysis of the structural, optical effects, and ASE characteristics in CsPb(Br_0.5_Cl_0.5_)_3_ with variations in the surface passivation thickness by PMMA polymers; to optimize the thickness level, and to compares their properties and responses to those of a bare CsPb(Br_0.5_Cl_0.5_)_3_ surface and those of PMMA layers of other thicknesses. The optical absorption, with a calculation of the absorption coefficient and binding energy as a function in the surface passivation layer thicknesses, was investigated systematically. Finally, detailed studies show the dependence of ASE properties (threshold, linewidth, and photo-stability) on the surface passivation layer thickness, which can be modified to optimize their performance. In this work, the combination of thermal evaporation deposition and surface encapsulation leads to a low ASE threshold and high ASE photo-stability, which improves the light amplification efficiency of the active layer in optoelectronic devices.

## 2. Materials and Methods

### 2.1. Materials

Lead(II) bromide (PbBr_2_, 99.999% trace metals basis); cesium bromide (CsBr, 99.999% trace metals basis); lead(II) chloride (PbCl_2_, 99.999% trace metals basis); cesium chloride (CsCl, 99.999% trace metals basis) and polymethyl methacrylate (Mw = 120,000 g/mol) (PMMA) were purchased from Sigma-Aldrich (Saint Louis, MO, USA). All chemicals were used as received, without further purification. 

### 2.2. Synthesis of CsPb(Br_0.5_Cl_0.5_)_3_ Thin Films and PMMA Thin Films

First, the glass substrates were cleaned and placed in a holder facing toward the precursor sources. Then, the CsPb(Br_0.5_Cl_0.5_)_3_ films were deposited onto the substrate through single-source thermal evaporation from the mixture of the primary components CsX and PbX_2_ (X = Br, Cl), with an equal stoichiometric ratio. The precursors materials were charged sequentially in a single boat and summarized in the reaction

$$PbBr_{2}+PbCl_{2}+CsBr+CsCl\to CsPbBr_{3}+CsPbCl_{3}\to 2CsPb{\left(Br_{0.5}Cl_{0.5}\right)}_{3}$$

**Figure 1:**
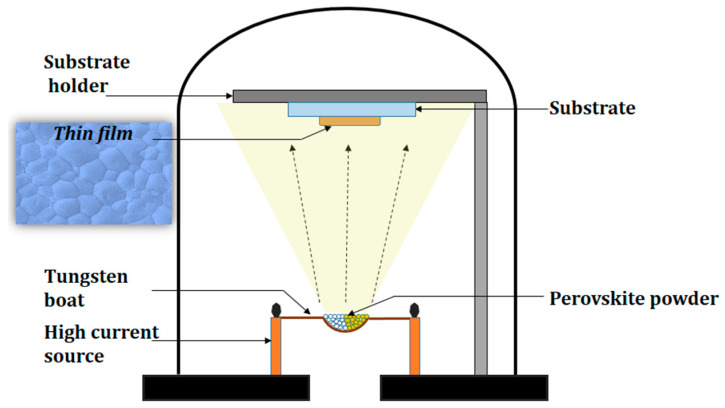
Schematic diagram of the thermal evaporation system.

After the evaporator chamber pressure was reduced to 10^−4^ mbar in the event of a few collisions with ambient gas molecules, both precursors were evaporated into the substratum by increased tungsten boat current and evaporation, as shown in Figure 1. 

To modify the perovskite surface for encapsulation, the PMMA thin films were prepared using a combination of thermal evaporation and spin-coating procedures. For the thermal evaporation coating procedure, a different quantity of the PMMA powders was charged in the boat at each stage to obtain the required thickness for the CsPb(Br_0.5_Cl_0.5_)_3_ films. The content of the PMMA was a tuning factor for controlling the film thickness. The PMMA films thicknesses were varied and adjusted to 0, 50, 100, 200, 300, 400, and 500 nm, with an error of less than 10%. However, control of the deposition rates is required to ensure that the final thin film consists of the correct stoichiometry in perovskite films and the PMMA thickness. The films were annealed at 120 °C for 15 min.

For the spin-coating procedures, toluene solutions containing PMMA (50 mg/mL) were prepared as stock solutions. Next, 100 µL of the PMMA stock solution was dropped onto the CsPb(Br_0.5_Cl_0.5_)_3_ films. The CsPb(Br_0.5_Cl_0.5_)_3_ films were placed in the spin-coating machine (4,000 rpm for 30 s) to prepare the homogenous thin films. After that, the coated films PMMA/CsPb(Br_0.5_Cl_0.5_)_3_, films were placed on a hotplate set to 100 °C for 30 min in ambient air. Finally, for PMMA-thickness measurements in every stage, pure PMMA films were condensed onto the clean glass substrate by thermal evaporation of the quantities of the PMMA powders and the same thing in the spin-coated procedure. 

Finally, to study the sample reproducibility, six samples from (PMMA film, t = 100 nm) were prepared similarly under the same conditions to compare their fluctuation range later.

### 2.3. Film Characteristics

Structural Characterization: All measurements were recorded at room temperature in an ambient environment. The morphology was investigated by using scanning electron microscopy (SEM) (JEOL-7600F, JEOL, Japan) and energy dispersive X-ray spectroscopy (EDS), (INCA-Exact Oxford system) was used for Elemental Analysis. The crystal phases of the films were characterized by X-ray diffraction (XRD). XRD was performed on an X-ray diffraction system (Miniflex 600, Rigaku, Japan) with copper Kα radiation (λ = 1.5418 Å). The scanning angle (2θ) varied between 10° to 80° at a scanning rate (step size) of 0.02° at 3°/min^−1^. 

Optical Characterization: For optical characterization, the absorption spectra of the samples were recorded by an ultraviolet-visible (UV–vis) spectrophotometer (V-670, JASCO, Tokyo, Japan). The PL spectra of the perovskite films were obtained by using a fluorescence spectrophotometer (FP-8200, JASCO, Tokyo, Japan) in the wavelength range of 350–800 nm. The film thickness was measured using a Dektak 150 stylus profiler (Bruker, Billerica, MA, USA).

ASE Measurement Experiments: To investigate the ASE and lasing properties of the films, power-dependent ASE spectra were collected at the sample edges near the ends of the excitation strips using the third harmonic generation LOTUS II Q-switched Nd: YAG picosecond laser (LOTIS, Minsk, Belarus) for excitation with a pulse duration of 70–80 ps at a repetition rate of 15 Hz. The signals emitted by the samples were directed through an optical fiber and a collimating lens. A QE65 Pro spectrograph (Ocean Optics, Dunedin, FL, USA) with a spectral resolution of 0.78 nm was used to detect all ASE signals and measurements. All measurements were taken while exposed to air at room temperature (293 K). 

Then, the pumping laser beam was passed through a plate with an aperture of ~2 mm in diameter. The laser beam was focused to a radius of ~2 mm when using the reflective microscope objective lens with a focal length of 10 cm. The signals emitted by the samples were directed through an optical fiber. The pumping laser energy density was adjusted by using a variable neutral density filter wheel. The energy of the laser was measured using an LM-P-209 coherent thermal sensor head. The laser energy density was attenuated to evaluate the threshold dependency on energy density.

Time-Resolved PL Measurements: Time-resolved PL measurements were performed using the third harmonic (355 nm) of a pulsed laser to excite the samples. Special filters were used to collect the laser excitation pulse from the detector and to select the wavelength emitted from the sample. The lens was used to collect and focus emission light onto a sensitive-enough fast photodiode (APD110). The fast Tektronix TDS 380 two-channel digital real-time oscilloscope (bandwidth 400 MHz, sample rate 2GS/s) was operated in a single hot mode to measure the time-scale decays and for data output. 

Data Analysis: A custom Python-based program developed by our research group was used to analyze the large amount of data that was collected in our study. The (peak-fit) program enabled us to rapidly obtain Gaussian fits of dual PL emission peaks in multiple data files.

## 3. Results

### 3.1. Structural Characterization

**Figure 2:**
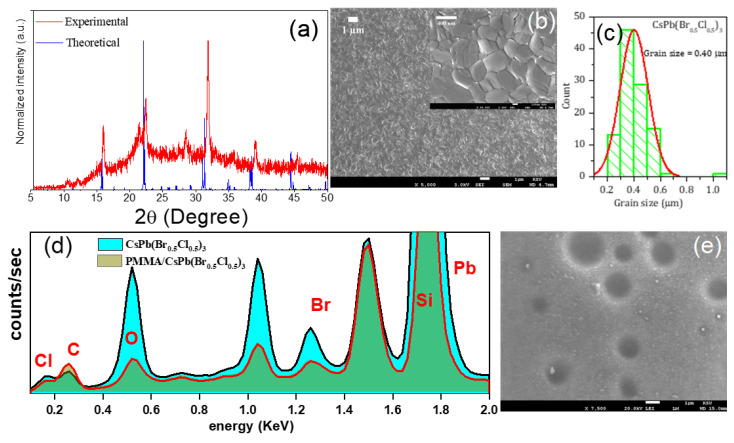
(**a**) X-ray diffraction (XRD) patterns. (**b**) Scanning electron microscopy (SEM) image of CsPb(Br_0.5_Cl_0.5_)_3_ perovskite thin films (scale bar, 1 µm), the inset is SEM image with high magnification (scale bar, 400 nm). (**c**) Histogram of the calculated grain size and Gauss fitting curves of the statistical data shown in the SEM image. (**d**) Energy-dispersive X-ray spectroscopy (EDS) spectra for perovskite bare and modified by PMMA polymer. (**e**) SEM image of perovskite film modified by PMMA layer (scale bar, 1 µm).

Figure 2a shows the experimental and theoretical XRD patterns of the CsPb(Br_0.5_Cl_0.5_)_3_ thin film. The theoretical XRD patterns were calculated using VESTA software [39]. The XRD pattern shows several diffraction peaks, specifically, peaks at 15.97°, 22.42°, 28.49°, 31.86°, and 39.06°. At room temperature, the CsPb(Br_0.5_Cl_0.5_)_3_ perovskite thin film has an orthorhombic structure in space group Pnma [40,41,42,43,44,45,46,47,48,49]. These theoretical data have appeared in consistency to experimental data.

The SEM image has analyzed the morphology of the CsPb(Br_0.5_Cl_0.5_)_3_ perovskite thin films. The thin film that was prepared using a thermal evaporation system was found to be uniform across the entire substrate, highly crystalline, pinhole-free, and composed of 0.40-μm-sized grains, as shown in Figure 2b,c. The energy-dispersive X-ray spectroscopy (EDS) spectrum, Figure 2d, gave us evidence that the perovskite film was coated with PMMA. According to EDS analyses, the spectra confirmed the presence of carbon and oxygen in both samples analysis. Since the composition of the perovskite bare film does not contain oxygen (O_2_) or carbon (C), so its presence in the EDS analysis can be attributed to CO_2_ contamination in the air. EDS result also showed a reduction in the counts of Cs, Pb, Br, and Cl in films which were coated by PMMA whereas showed an increase of C peak. In the perovskite film, carbon was achieved from CO_2_ contamination in the air. whereas the carbon from PMMA corresponding to the higher CO_2_ concentration of air. Figure 2e showed that the PMMA is sensitive to electron beams, resulting in damage of the PMMA trenches, which is in agreement with the previous report [50]. Therefore, the SEM images in the films modified by PMMA do not give information on the PMMA thickness by SEM cross-section. Also, the sample preparation cross-sectional images will damage the edge of the top surface of PMMA. So, the suitable technique to estimate the thickness of the film by using a surface profilometer (Veeco Dektak 150) rather than measuring the film thickness by SEM cross-section. The PMMA layer thickness was measured to 0, 50, 100, 200, 300, 400, and 500 nm, with errors less than 10% thickness variation over the whole film.

### 3.2. Optical Characterization

#### 3.2.1. Absorption and Steady-State Photoluminescence Measurements

**Figure 3:**
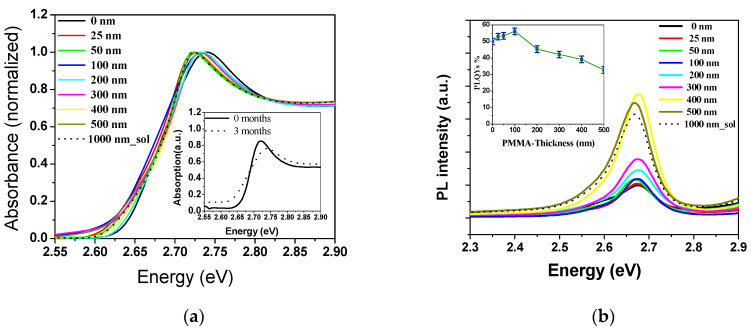
(**a**) Optical absorption spectra, the inset is the time-dependent absorption spectra for CsPb(Br_0.5_Cl_0.5_)_3_ perovskite film; (**b**) emission spectra of CsPb(Br_0.5_Cl_0.5_)_3_ thin films, bare and with various coating thicknesses of polymethyl methacrylate (PMMA) as a passivation layer, the inset is the PMMA thickness-dependent photoluminescence quantum yield (PLQY). Modulus error bars are ±2-unit standard deviation.

The optical absorbance spectra of the thin films were recorded in the UV–vis region of the electromagnetic spectrum, as shown in Figure 3a. The absorption onset can be observed at ∼2.78 (447 nm) for all samples, which corresponds to the near band edge of CsPb(Br_0.5_Cl_0.5_)_3_ regardless of the thickness of the PMMA. The absorption comprises three broad features: a sub-bandgap absorption tail at low energy, followed by a strong exciton peak, then band-to-band transitions [51,52]. Broad absorption spectra with an exciton peak position at between 2.702 and 3.03 eV were observed for evaporation, while the PMMA thickness peaked between 0 and 500 nm and was spin-coated to 1000 nm film thickness. Storing the film under ambient conditions causes the CsPb(Br_0.5_Cl_0.5_)_3_ thin film to exhibit an 11% drop in the PLQY after 3 months (inset, Figure 3a). This indicates the necessity of the encapsulation of perovskite film. In terms of the initial, the blue shift (13 MeV) was presented by the time-dependent absorption. The blue shift and photobleaching of perovskite can be attributed to the irreversible photo-oxidation of perovskite [53].

Next, the room-temperature PL properties of the films were investigated at λ_ex_ = 355 nm. The PL peaked around 2.693 eV for CsPb(Br_0.5_Cl_0.5_)_3_ bare film, and its full-width at half-maximum (FWHM) was 93.72 meV, as shown in Figure 3b. Moreover, as shown in Figure 3b, by increasing the thickness of the polymer from 0 to 500 nm, the PL FWHM and its peak position fluctuated between (2.669 and 2.693 eV) and (80.98 and 97.00 eV), respectively. These lower FWHM values suggest superior quality and good optical properties of the fabricated thin films. All the PL parameters were tabulated as shown in Table 1.

A correlation between the PL efficiency of the CsPb(Br_0.5_Cl_0.5_)_3_ and the PMMA film thickness was revealed. For CsPb(Br_0.5_Cl_0.5_)_3_, the photoluminescence quantum yield (PLQY) in the toluene solution reached up to 50% by taking R6G as a reference dye (Φ_R_ = 0.95 in ethanol). Whereas for modified films by PMMA layer, PLQY ($\phi_{S}$) was measured for three samples tested per trial by comparing to the perovskite bare film through the equation

$$\phi_{S}=\phi_{R}\frac{A_{PL_{S}}}{A_{PL_{R}}}\;\cdot \;\frac{I_{ABS_{R}}}{I_{ABS_{S}}}$$

With the addition of a PMMA, the PL efficiency initially increased, exhibiting a maximum PL efficiency of 56% at 100 nm. An excessive amount of PMMA coating could be the origin of PL efficiency drops at higher thicknesses. A thicker coating significantly deteriorated the PL efficiency to 32% at t = 500 nm (inset Figure 3b). We also confirmed high PLQYs by studying the ASE differential quantum efficiency (to be described later). Therefore, the highest PLQY resulted in the lowest ASE threshold. Although the photo-stability increases with the PMMA thickness, the PL efficiency is sacrificed at high PMMA thickness as well as in optical absorption coefficient.

**Figure 4:**
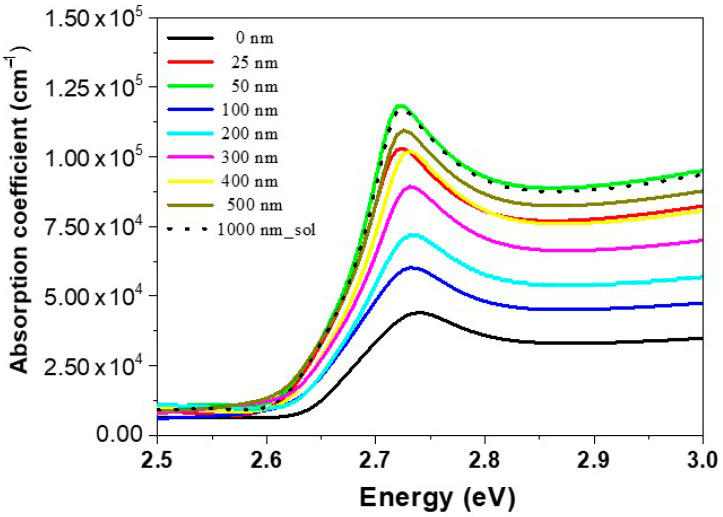
Absorption coefficient spectra of CsPb(Br_0.5_Cl_0.5_)_3_ thin films, bare and with various coating thicknesses of polymethyl methacrylate (PMMA) as the passivation layer.

The optical absorption coefficient (*α*) was calculated from the optical absorption spectra using the formula $\alpha =\left(\frac{2.303\;A}{t}\right)$, where *t* denotes the CsPb(Br_0.5_Cl_0.5_)_3_ film thickness (400 nm), and *A* is the absorption spectra. Therefore, the α for the optical absorption spectra could be obtained by using the absorption spectra, as shown in Figure 4. The magnitude of α increases as the polymer thickness increases. Parameter α shows high values (10^5^ cm^−1^), which indicates an improvement in the layer stacking of the films by evaporation; i.e., the closer the atoms are to each other, the more disordered the atoms become and the greater the reduction of the number of vacancies, which results in a high α value [54]. As mentioned in our results, the PMMA does not affect the optical and structural properties of perovskite due to its transparency and amorphous structure. To study the effect of PMMA film thickness on optical performance, absorption coefficient spectra of nine films (without PMMA, 25, 50, 100, 200, 300, 400, 500, and 1000 nm) are plotted in Figure 4. Compared with the film of PMMA-free (t = 0 nm), absorption coefficient enhancement across almost the whole waveband for films with PMMA coating can be observed. With the PMMA thickness increasing from 0 to 25 nm, the absorption coefficient increase, then decrease dramatically at t = 100 nm. The more increase in PMMA thickness after that, the absorption coefficient will return to increase. With the PMMA thickness increasing from 200 to 1000 nm, the absorption coefficient increase and absorption peaks red-shifted, leading to an increase in the optical losses, which is reflected in the ASE threshold results. Thus, 100 nm-thick PMMA is more suitable to reduce absorption losses and reduce the ASE threshold at the same time with plays the role of both surface passivation and symmetric waveguides. Also, the optimum thickness could maintain the optical property and endow perovskite film with high stability. Low ASE threshold in perovskite films with the PMMA layer may shed light upon the development and application of perovskite stable and sustained lasers. The increase in PMMA film thickness causes increasing film scattering with film thickness which is reflected in the PL spectra (Figure 3b). The PL results do not show the optimum thickness due to the absorption in the surface layer on the top of the perovskite film, which could be removed by the higher energy pulses (laser). As discussed in the ASE section.

The calculated *E*_g_ was performed by evaluating the Coulomb field of the exciton which is known as the Summerfield factor *S*(*E*), where *E* represents photon energy [51]. Here, we have taken into account the *S*(*E*) to evaluate the absorption coefficient *α*(*E*) for the films by using the following expression [51,55,56]

(1) α(E)=X+conv (Y×Z)

where

(2) X=(22π)×(Eb)32×Aσ1exp(−(E−Eg−Eb)22 σ12)

(3) Y=(22π)×(Eb)32×Bσ2exp(−(E−Eg−Eb)22 σ22)

(4) Z=(2π×C×(E−Eg)12× θ(E−Eg))×S(E)

(5) $$S\left(E\right)=\left(\frac{2\pi \;\sqrt{\frac{E_{b}}{E-E_{g}}}}{1-\exp(-2\pi \;\sqrt{\frac{E_{b}}{E-E_{g}}}}\right)$$

where A, B, and C are constants. The quantities *E*_b_ and *E*_g_ are the exciton-binding energy and energy bandgap, respectively; *σ*_1_ and *σ*_2_ are the FWHMs of the excitonic peaks. Conv(*Y*×*Z*) and $\theta \left(E-E_{g}\right)$ represent the convolution of the functions (*Y*, *Z*) and the Heaviside step function, respectively. In Equation (4), the first term represents the exciton transition, where the exciton peak was considered to have a Gaussian peak shape with a width of *σ*_1_; and the second term represents the band-to-band transitions, including the *S*(*E*) factor.

**Figure 5:**
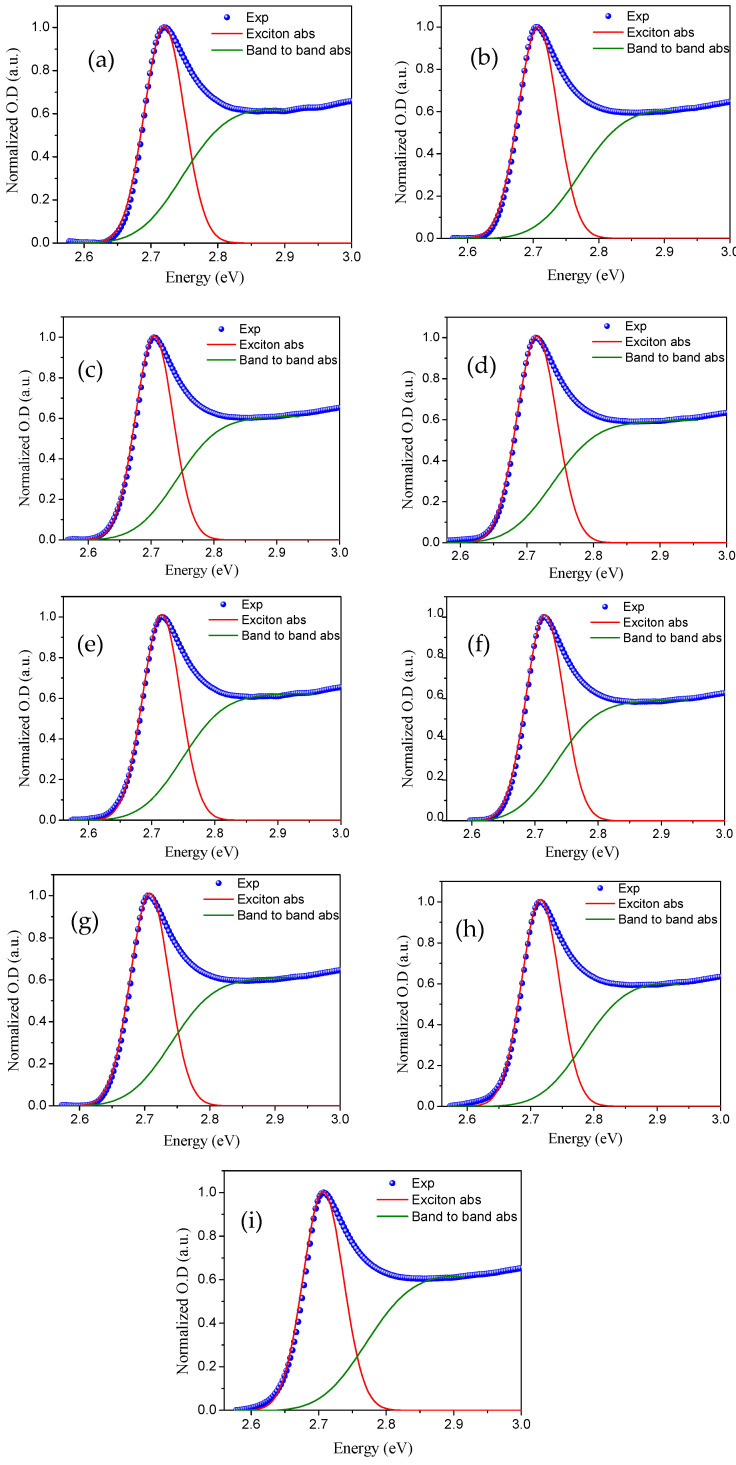
Excitonic and band-to-band absorption with experimental data for CsPb(Cl_0.5_Br_0.5_)_3_ with a polymethyl methacrylate (PMMA) thickness of (**a**) 0 nm, (**b**) 0 nm_sol, (**c**) 25 nm, (**d**) 50 nm, (**e**) 100 nm, (**f**) 200 nm, (**g**) 300 nm, (**h**) 400 nm, and (**i**) 500 nm.

Figure 5 shows the excitonic and band-to-band absorption for all of the investigated thin films. The calculated *E*_b_ values varied between 67.00 and 75.30 meV, whereas the *E*_g_ value remains nearly the same for all films, which indicates that the polymer thickness has no major effect on the electronic properties of perovskite materials.

**Figure 6:**
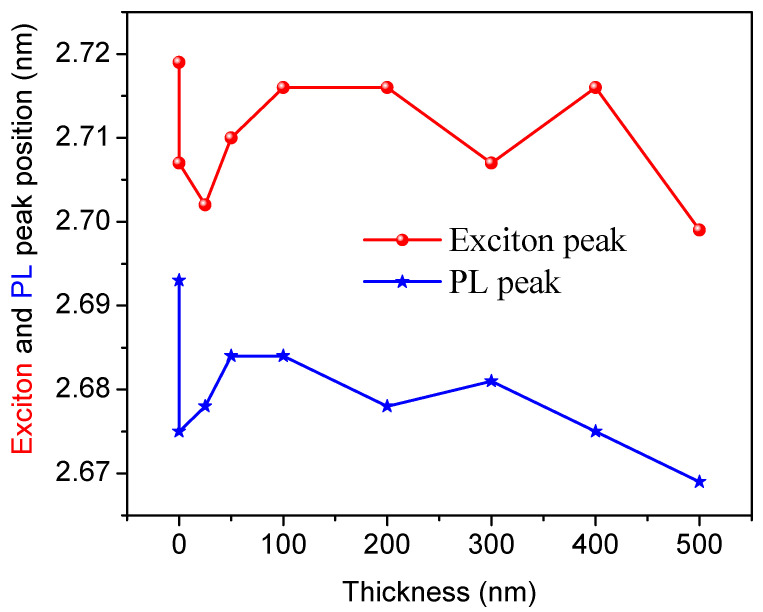
Exciton and photoluminescence (PL) peak positions of perovskite thin films versus the polymethyl methacrylate (PMMA) thickness.

The optical band gap—e.g., exciton and PL peak positions, FWHMs of the exciton, and PL of the perovskite films—are tabulated in Table 1 and Figure 6.

#### 3.2.2. Time-Resolved PL Studies

**Figure 7:**
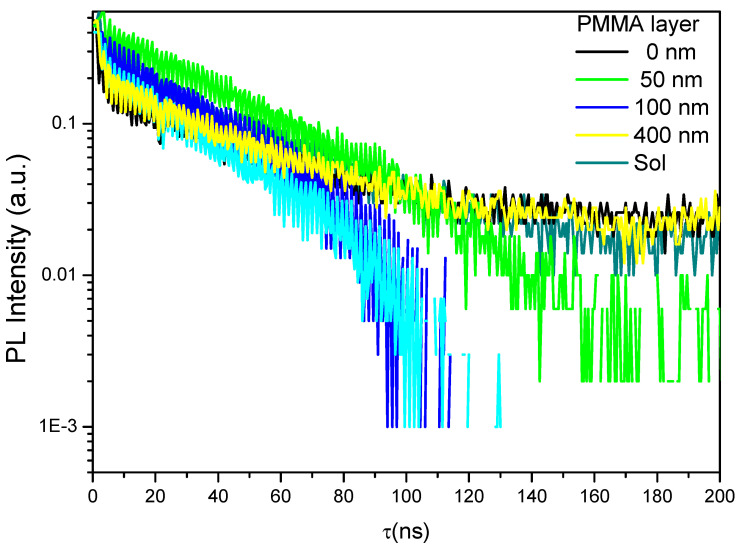
Time-resolved photoluminescence (PL) of CsPb(Br_0.5_Cl_0.5_)_3_ thin films, bare and with various thicknesses of polymethyl methacrylate (PMMA) as a passivation layer.

To further understand the role of the mechanism of surface passivation, time-resolved PL studies were conducted under low laser fluence to avoid saturation effects. After the laser excitation pulses, the bands flatten immediately, and changes in the injection PL signal are likely due to variations in the capture cross-section of surface traps. For our analyses, we created photo-generated carriers with the appropriate penetration depth for the sample, which we were then able to diffuse, recombine radiatively and non-radiatively in the bulk, and then recombine non-radiatively. Representative time-resolved PL data for several different surface passivation layer thicknesses are shown in Figure 7. While none of the PMMA surface passivation layers yielded PL decay times that lasted as long as those observed for the bare CsPb(Br_0.5_Cl_0.5_)_3_ perovskite film. The effects of surface passivation clearly produced an interface with a lower surface recombination velocity than that obtained from the un-passivated air-exposed surface. The recombination time reduces with an increase in the PMMA thickness until 200 nm, after which the recombination time increases but not longer than that for the bare CsPb(Br_0.5_Cl_0.5_)_3_ perovskite film. These data clearly indicate that increases in the steady-state PL signal do not necessarily correlate with decreases in the surface recombination rates under high injection conditions.

### 3.3. ASE Measurements

The stimulated ASE process serves as a crucial and fundamental step for evaluating the suitability of a material for gain enhancement when pumped to produce a population inversion, thereby achieving a cavity-free configuration. The feedback made by ASE via the optical cavity is necessary to produce a laser operating at a lasing threshold. Thus, ASE provides an applicable benchmark for determining the material suitability for gain applications. The coherence degree of the laser is much higher than that of ASE, even though the ASE may be much more intense [28,57].

#### 3.3.1. ASE Pump Fluence-Dependent

As mentioned in the introduction section, it is commonly believed that efficient light emission from CsPbX_3_ perovskites can only be achieved with nanoparticles. Herein, we perform an analysis of the ASEs properties –under picosecond laser as excitation source at λ_ex_ = 355 nm—to examine the role of surface passivation based on the various thicknesses of the PMMA polymer coating and compared their properties and optical response to those of the bare perovskite CsPb(Br_0.5_Cl_0.5_)_3_ films in the bulk phase. The PMMA is a transparent polymer with a non-crystalline structure. Moreover, refractive indices in a polymer are appreciably different from perovskite at a wavelength that perovskite emission uses by 13% to enables a sizable bandwidth. 

**Figure 8:**
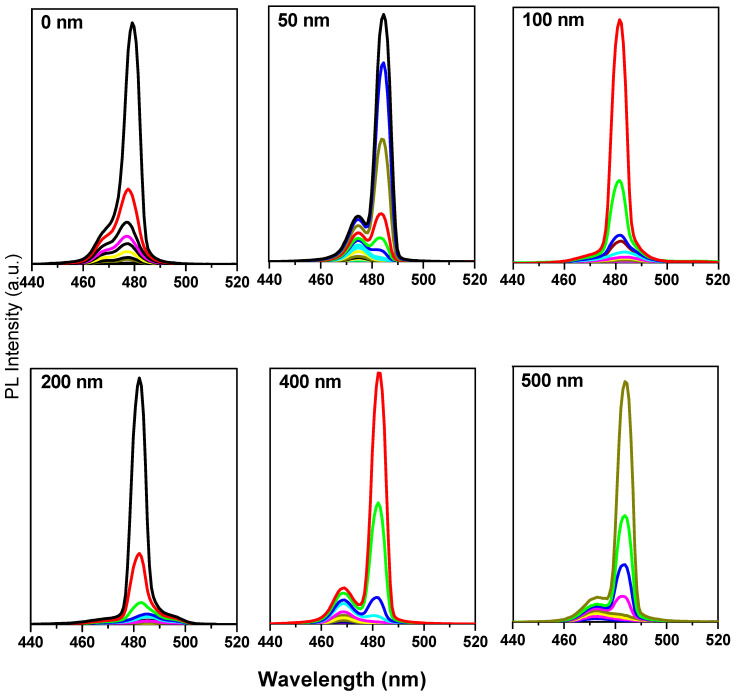
Pump-fluence relationship for CsPb(Br_0.5_Cl_0.5_)_3_ thin films, bare and with various thicknesses of polymethyl methacrylate (PMMA) polymer coating as a passivation layer.

The ASEs properties and a comparison of the ASE performance of PMMA-modified CsPb(Br_0.5_Cl_0.5_)_3_ surfaces of various thicknesses with the bare CsPb(Br_0.5_Cl_0.5_)_3_ surface films were determined as follows. As can be seen in Figure 8, the ASEs were generated by an increase in the pump fluence in CsPb(Br_0.5_Cl_0.5_)_3_ at the bulk structure in both bare films and surfaces modified with a passivation layer of a PMMA polymer coating of various thicknesses. At low pump energy, the PL spectrum is broad and featureless; however, as the pump energy increased above the ASE threshold, the narrower peak that appears at the point of redshift indicates the occurrence of an ASE state.

In bare CsPb(Br_0.5_Cl_0.5_)_3_ thin films, ASE generated at 473 nm shifted to 480 nm. The onset of the stimulated emission was observed to have an immediate increase in ASE intensity, with a narrowing of the emission spectrum (FWHM ≈ 6 nm) over the threshold pumping range. A redshift in the ASE peak (7 nm) relative to the PL peak was observed.

For CsPb(Br_0.5_Cl_0.5_)_3,_ thin films modified by the passivation layer and for all PMMA thicknesses, a stimulated emission was observed to have an immediate increase in the ASE intensity, with a narrowing of the emission spectrum (FWHM ≈ 6 nm) over the threshold pumping range, with differed only for the ASE pumping threshold. Then, ASE generated at 474 nm shifted to 484 nm relative to the PMMA thickness (t = 25, 50, and 100 nm). For PMMA thicknesses greater than 100 nm (when t = 200, 300, 400, 500, and 1000 nm (produced from a solution)), ASE generated at 474 nm shifted to 482 nm.

The reabsorption effect, which appeared at the overlap of the absorption band edge with the PL emission (spontaneous emission spectrum) suggests that the self-absorption effect should take place in the ASE state.

The pumping energy density threshold of ASE was determined by the simultaneous occurrence of spectral narrowing of the emission and the ASE peak near the long-wavelength region of the spontaneous emission, which is followed by rapid growth. Furthermore, the threshold is estimated by determining the mean value of the minimum pump energy density that allows for the ASE regime to be measured in three different positions on the sample.

The ASE linewidth or FWHM for all samples decreased as the pumping energy density increased. The FWHM ASE was saturated above the ASE threshold value. The PL spectra show two peaks that facilitate the proper determination of FWHM, which is difficult to determine by the normal calculation method. Therefore, we used the peak-fit program to fit the expansive data.

**Figure 9:**
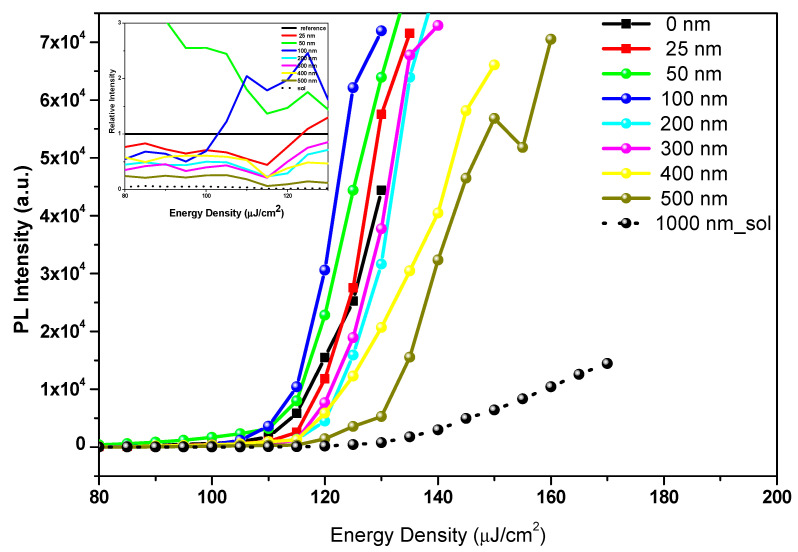
Photoluminescence (PL) intensity versus the energy density of CsPb(Br_0.5_Cl_0.5_)_3_ thin films, bare and with various thicknesses of polymethyl methacrylate (PMMA) as a passivation layer. The inset figure shows the relative intensity of various thicknesses of the passivation layer to bare film for t = 0, which is used as a reference for the energy density.

In another method, the threshold can also be determined by the intensity at which a change of slope causes a nonlinear increase in the emission intensity when it is observed within an output versus input intensity curve. A plot of the peak intensity versus the pump energy density (Figure 9) shows that a sharp rise occurred at approximately the threshold, which indicates the transition from spontaneous to stimulated emission.

It should also be noted that thresholds determined from the change of slope are slightly higher than those obtained from the FWHM decay because the change-of-slope takes place when the spectrum is already narrow. Spectral narrowing generally results from ASE due to stimulated emission.

#### 3.3.2. ASE Properties (Threshold, Linewidth, and Photo-Stability) Versus PMMA Film Thickness

**Figure 10:**
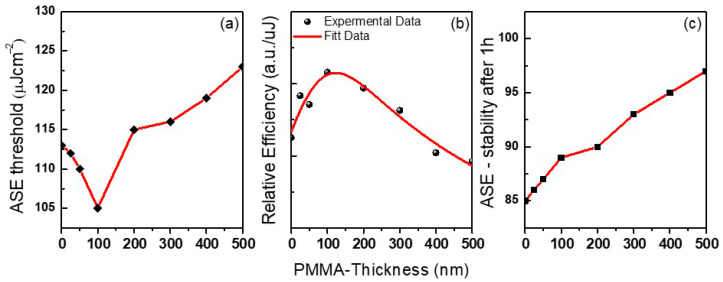
(**a**) Amplified spontaneous emission (ASE) energy density threshold; (**b**) relative efficiency of fitted lines beyond the threshold in intensity-pump energy depending on the curves as a function of the energy density; and (**c**) ASE intensity under laser shot for 1 h (54,000 shots) as a function of the thicknesses of polymethyl methacrylate (PMMA) as a passivation layer top of CsPb(Br_0.5_Cl_0.5_)_3_ thin films.

A plot of the PMMA film thickness (t) versus the ASE threshold is shown in Figure 10a, which suggests the lasing threshold is dependent on multiple parameters involving t. The increase in the ASE threshold for thicknesses greater than t = 100 nm can be attributed to the reduced confinement causing a decrease in the ASE quality due to an increase in the leakage signal. Surface smoothing will decrease the loss of incident pumping light at the interface of the air/perovskite thin film, which will lead to a higher level of emission and achieve high-performance light emission, which may enhance its performance when used in the creation of ASE, lasing applications, thin films, and active-layer photonics devices. As was observed, the lowest ASE threshold was obtained at t = 100 nm. Concerning t = 100 nm, films showed better performance with a value of 102 µJ/cm^2^, in contrast with the 113 µJ/cm^2^ and 115 µJ/cm^2^ obtained for t = 0 nm and t = 200 nm, respectively. The ASE threshold arrival to 123 µJ/cm^2^ at t = 500 nm.

The time-resolved PL results were first ascribed to the passivation of defects in the perovskite top surface, accounting for the higher PL intensity and the slower PL relaxation, as well as for approximately 9% of the ASE threshold decrease. The remaining ASE threshold decrease was instead ascribed to improved waveguiding, which was facilitated by the realization that an almost symmetric glass-perovskite-PMMA waveguide results in higher mode confinement in the perovskite layer concerning the asymmetric glass–perovskite layer. 

The PL slope versus the energy density curves in the ASE region is an indicator of the quantum efficiency of the ASE process. The slopes (deduced from the slope of the PL intensity versus energy density curves (Figure 9)) were calculated and plotted in Figure 10b for CsPb(Br_0.5_Cl_0.5_)_3_ thin films, bare, and with various thicknesses of PMMA as a passivation layer. Also, it was found that the differential quantum efficiency increases when the thickness is approximately 100 nm.

ASE demonstrated continuous operational stability against photo-degradation and optical gain property characteristics in CsPb(Br_0.5_Cl_0.5_)_3_ thin films. Generally, photo-stability is evaluated by recording the total ASE intensity emitted under a constant pump intensity just above the threshold, as a function of time. The presence of photo-degradation is identified when a decrease in the total ASE output is observed. Figure 10c. shows the results that were obtained for films of various PMMA thicknesses. As was observed, the best results were obtained for t = 500 nm, which showed an ASE lifetime of 54,000 pulses (i.e., 60 min) for the perovskite film. 

Finally, The photo-stability increases with the PMMA thickness. As a result, the stability is enhanced, but the PL efficiency is sacrificed after the treatments (inset Figure 9). It is necessary to highlight here that applying protection materials is a function of both surface passivation and encapsulation by a single-step treatment in a single protection process, which with synergetic effects could maintain the optical property and endow perovskite film with high stability.

### 3.4. Sample Reproducibility

**Figure 11:**
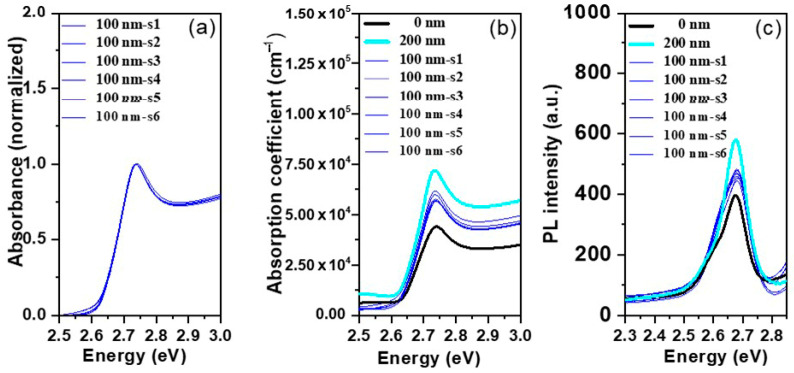
(**a**) Optical absorption spectra; (**b**) absorption coefficient spectra; (**c**) emission spectra of six samples (CsPb(Br_0.5_Cl_0.5_)_3_ perovskite thin films) encapclated by PMMA film, t = 100 nm, compared to samples with a thickness of PMMA layer (t = 0 and 200 nm).

Samples reproducibility reflects the consistent degree of the results. Therefore, for proving the sample reproducibility, six samples from (PMMA film, t = 100 nm) were prepared similarly under the same conditions and observe their absorbance, PL performances, and absorption coefficient spectra of the thin films. Then these results for six samples (t = 100 nm) were compared with the results in Figure 3b and Figure 4 for t = 0 and 200 nm and plot it in Figure 11. 

**Figure 12:**
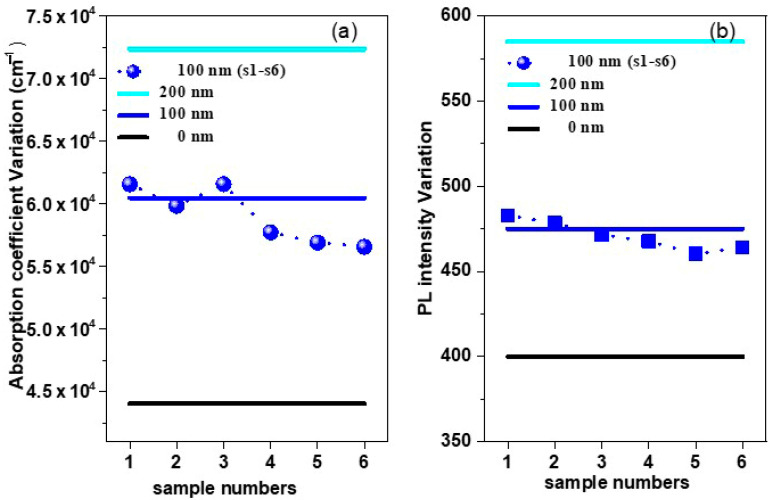
Intensity fluctuation of (**a**) absorption coefficient and (**b**) PL intensity fluctuation of six samples analyzed.

Figure 12 shows the variation in the intensity fluctuation of the absorption coefficient and PL Intensity of six samples analyzed (PMMA film, t = 100 nm), which deduced from Figure 11b,c. The observed fluctuation for the absorption coefficient and PL Intensity in the range of 6.46% and 3.16%, respectively. In general, the fluctuation of the results exhibited in a range of less than 7% which less than the error in the film thickness variation over the whole film besides the other experimental errors. 

The reproducibility of our results was ascertained by depicting the optical response derived from different measurements for a batch of six samples (Figure 11 and Figure 12) which were showed the observed fluctuation range is less than the effect of PMMA film thickness. 

Finally, the results obtained were representative and the observed data fluctuation neglect compared to the effect on PMMA layer thickness. The major effect of the PMMA layer thickness on the absorbance and PL performance is consistent with what was reached in previous reports [34,58].

## 4. Conclusions

Encapsulation of the top layer of perovskite films was achieved in a single-source thermal evaporation system by using PMMA as a specialized surface passivation approach. The effects of the surface-passivation layer and its thickness on the optical response were enhanced the ASE properties. The achieved ASE from the bulk structures of CsPb(Br_0.5_Cl_0.5_)_3_ confirms that efficient light emission can be achieved from sources other than CsPbX_3_ perovskites nanoparticles. Our results demonstrated an important impact on achieving blue light emissions from perovskite as a bulk structure. Then, analysis of the ASE properties revealed the role of various thicknesses of PMMA polymer coatings in surface passivation by comparing their properties and optical response to bare perovskite CsPb(Br_0.5_Cl_0.5_)_3_ films. In the surface passivation approach, the surface-modification-dependent optical properties can suppress surface states and improve surface quality without optical or structural changes. Surface passivation resulted in enhanced ASE, which is key to improving the performance of optoelectronic devices. Key ASE characteristics—threshold, linewidth, emission wavelength, ASE growth, and photo-stability—appeared to initially change based on the surface passivation layer thickness, with the best results achieved when the surface passivation layer was 100 nm thick. As a result, stability was enhanced with the PMMA coatings of various thicknesses, but the PL efficiency was sacrificed when the thickness was greater than 100 nm. Reproducibility of our results mention that the 100 nm-thick PMMA is more suitable for reducing absorption losses and reducing the ASE threshold. Also, the optimum thickness could maintain the optical property and endow perovskite film with high stability. Finally, our results confirmed that the PMMA layer plays the roles of both surface passivation and symmetric waveguides. Low ASE threshold in perovskite films with the PMMA layer may shed light upon the development and application of perovskite stable and sustained lasers. 

## Figures and Tables

**Table 1:** Optical band gap e.g., exciton and photoluminescence (PL) peak positions, full-width at half-maximum (FWHM) of the exciton and photoluminescence (PL) of CsPb(Br_0.5_Cl_0.5_)_3_ thin films, bare and with various thicknesses of polymethyl methacrylate (PMMA) as a passivation layer.

Thickness (nm)	Bandgap Eg (eV)	Exciton Binding Energy Eb (meV)	Exciton Peak (eV)	FWHM Exciton Peak (meV)	PL Peak (eV)	Stock Shift (meV)	FWHM of PL Peak (meV)
0	2.787	67	2.719	70.67	2.693	26	93.72
25	2.775	70	2.702	70.65	2.678	24	81.69
50	2.785	70	2.71	70.72	2.684	26	96.81
100	2.786	70.2	2.716	70.67	2.684	32	95.82
200	2.786	70.2	2.716	70.67	2.678	38	87.27
300	2.778	70.2	2.707	70.65	2.681	26	80.98
400	2.786	70.2	2.716	70.67	2.675	41	97.00
500	2.782	75.3	2.699	70.68	2.669	30	81.56
1000_sol	2.774	66.8	2.707	70.66	2.675	32	85.86
